# Intervention on whole grain with healthy balanced diet to manage childhood obesity (GReat-Child™trial): study protocol for a quasi-experimental trial

**DOI:** 10.1186/s40064-016-2431-y

**Published:** 2016-06-22

**Authors:** H. C. Koo, B. K. Poh, Abd Talib Ruzita

**Affiliations:** Nutritional Sciences Programme, School of Healthcare Sciences, Faculty of Health Sciences, Universiti Kebangsaan Malaysia, Jalan Raja Muda Abdul Aziz, 50300 Kuala Lumpur, Malaysia; Department of Healthcare Professional, Faculty of Health and Life Sciences, Management & Science University, Shah Alam, Malaysia

**Keywords:** Behaviour change, Childhood obesity, Healthy eating, Intervention, Nutrition education

## Abstract

**Background:**

The rapid increase in childhood obesity is a serious public health problem, and has led to the development of many interventions. However, no intervention has emphasized whole grains as a strategy to manage childhood obesity. Therefore, this article describes the protocol of a 12-week multi-component, family-based intervention on whole grain, using a healthy balanced diet for managing childhood obesity.

**Methods:**

The GReat-Child trial utilize a quasi-experimental method in which two schools in Kuala Lumpur are assigned to intervention and control groups. The eligibility criteria are overweight/obese children, aged 9 through 11 years, who has no serious co-morbidities. The children who report consuming whole-grain foods in their 3-day diet-recall during the screening will be excluded. The study sample is characterized by anthropometric measurements (weight, height, percentage of body fat and waist circumference), whole grain and nutrient intakes (3-day 24-h diet recalls), and their knowledge, attitudes and practices towards whole grain. The 12-week intervention is comprised of three components addressing behaviour, personal and environmental factors, based on social cognitive theory: (1) individual diet counselling for the parents; (2) six 30-min nutrition education classes and (3) school delivery of whole-grain foods; The control school does not receive any interventions, however, for ethical purposes, a health talk is conducted after the entire GReat-Child Trial is completed.

**Conclusion:**

The GReat-Child trial represents a novel approach to examining the effectiveness of the intervention of whole grain in a healthy balanced diet on managing childhood obesity. We anticipate that this trial will reveal not only whether whole grain intervention will be effective in managing childhood obesity, but also provide greater insights into the acceptance of whole grain among Malaysian children.

## Background

Childhood obesity is a chronic disorder, which is defined as excessive body fat deposition that presents an adverse effect on health (Fernandez-Sanchez et al. 2011). It poses a global public health threat and has risen to an alarming level throughout the world (Theodore et al. 2009). The long-term persistence of obesity in childhood leads to several chronic diseases, such as hyperlipidaemia, hypertension and hyperinsulinemia (Linberg et al. 2012). Apart from the above-mentioned chronic diseases, the findings from one study have indicated a significant increase in the psychosocial consequences of childhood obesity (Centers for Disease Control and Prevention 2009). For example, children who are obese have a negative body image, which leads to lower self-esteem, and reflects negatively on their academic and social progress (Ben-Sefer et al. 2009). The complications caused by childhood obesity are severe, and could continue to affect the health of a child, even in adulthood (Centers for Disease Control and Prevention 2009).

In recent years, the rates of childhood obesity in Malaysia have been rising dramatically (IPH 2011), similar to the global patterns (Kipping et al. 2006). This rising problem of childhood obesity has led to the development of intervention programs against obesity starting in early life. Successful childhood obesity intervention may be useful as a secondary prevention, decreasing the complications of childhood obesity on co-morbidities later in life (Reilly 2006). The essential elements of any intervention are likely to include dietary modifications and nutritional education (Kirk et al. 2005). Several childhood obesity interventions testing nutritional education and diet modification have been initiated in several countries, such as the United States (Davison et al. 2013), Canada (Anand et al. 2007), the United Kingdom (James et al. 2007), New Zealand (Graham et al. 2008), Singapore (Gupta et al. 2010) and Malaysia (Sharifah et al. 2011); however, none of these interventions emphasized the provision of whole grain to manage childhood obesity.

A recent meta-analysis concluded that adults consuming more than three servings of whole grain had a consistently lower risk of obesity (Ye et al. 2012). Additionally, both epidemiological (McKeown et al. 2009) and randomized intervention trials (Maki et al. 2010) conducted among adults from the US have demonstrated that the abdominal fat mass was reduced on a whole-grain diet, compared to refined grain, an effect which has not been reported for fruits and vegetables (McKeown et al. 2009). In children, the correlation between the consumption of whole grain and health benefits has been less explored. However, previous cross-sectional studies from the US have reported that the consumption of whole grain can improve the diet quality (Bellisle et al. 2014) and body mass index (BMI) z-scores (Choumenkovitch et al. 2012) among children.

In spite of the positive health benefits and dietary recommendations, the national dietary intake data from the US (Harnack et al. 2003), France (Bellisle et al. 2014), Malaysia (Norimah et al. 2015) and Singapore (Health Promotion Board Singapore 2010) have demonstrated that whole grain consumption is considerably low. In Kuala Lumpur, a recent local study revealed that the practice of consuming whole grain was low there too, with less than 10 % reported daily consumption of whole grain (Koo et al. 2015a). A quasi-experimental trial has been conducted in the US, aiming to increase the whole grain consumption among children (Burgess-Champouxt et al. 2007). However, this quasi-experimental trial did not investigate the association of whole grain intake with anthropometric measurements, which helps in managing childhood obesity (Burgess-Champouxt et al. 2007).

Home environment and family has been widely recognized as one of the influences in child’ seating behaviours and dietary intake (Golan 2006). Parental involvement is a critical and feasible component in an intervention which raises positive dietary behavioural changes for children (Perry et al. 1998). A meta-analysis that involved 42 weight-related health interventions demonstrated that parental involvement in the interventions was more effective in managing childhood obesity (Niemeier et al. 2012). Another review revealed that the majority of studies reported a positive effect of home-based interventions which included family members in combating childhood obesity (Knowlden and Sharma 2012). The overall effectiveness and sustainability of an intervention program could be improved by identifying parent-friendly strategies that promote active, sustained participation (Burgess-Champouxt et al. 2007).

The purpose of this present article is to describe the rationale and design of a multi-component, family-based intervention on whole grain combined with a healthy balanced diet to manage childhood obesity, utilizing a quasi-experimental trial design: the GReat-Child Trial. To the best of our knowledge, this is the first trial that has focused on whole grain to manage childhood obesity. It was hypothesized that, in the school post-intervention comparison, the children in the intervention school would have: (1) improved anthropometric measurements by increasing their wholegrain consumption; (2) improved knowledge, attitudes and practices towards whole grain; (3) improved overall nutrient intake and (4) increased availability of whole-grain foods at home.

## Methods

### Trial design and sample size calculation

**Fig. 1:**
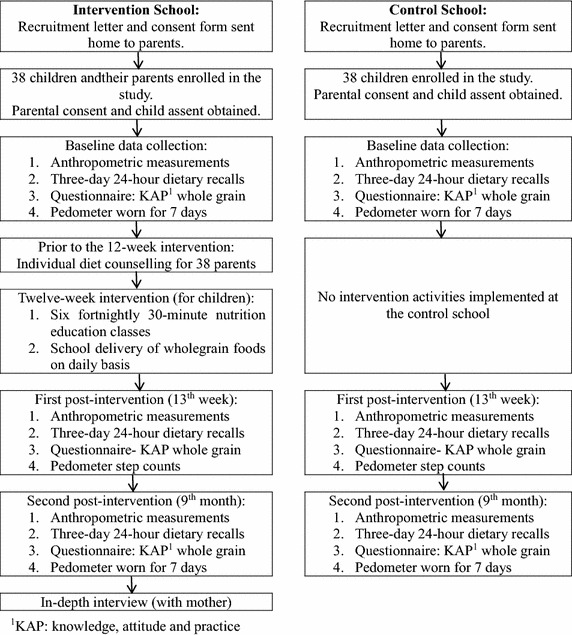
Flow diagram of the GReat-Child trial design

The flow diagram of the trial design is shown in Fig. 1, and a list of government primary schools in Kuala Lumpur is obtained from the Kuala Lumpur Federal Territory Education Department. Kuala Lumpur is the federal capital and most populous city in Malaysia, and consists of three zones: Sentul, Keramat and Bangsar-Pudu. Two primary schools are recruited from a randomly selected zone to participate in the present trial. Schools with similar social and demographic characteristics are assigned to the intervention and control conditions on a non-randomized basis, in order to make sure the intervention and control groups were completely independent, sufficiently far apart and without any effect on each other (McMillan 2007). The inclusion criteria are: (1) apparently healthy Malaysian school children aged 9–11 years or studying in years 4 and 5; (2) children who are overweight or obese (BMI for age > +1 SD, relative to the WHO reference data) (World Health Organisation (WHO) 2007); (3) able to read, write and understand Malay; and (4) at least one parent who perceives that their child has a weight problem, and who is willing to attend the individual diet counselling. The perception of childhood obesity is considered important in order to recruit only participants who are receptive to the intervention, and who are sufficiently motivated to complete all of the intervention sessions (Wafa et al. 2011). Children are excluded: (1) if they have a serious co-morbidity requiring treatment or on gluten-free diet and (2) if any of their 3-day 24-h diet-recall during screening indicate that they had consumed whole-grain food in the previous week. The study protocol was reviewed and approved by the Universiti Kebangsaan Malaysia Research Ethics Committee, and permission to carry out the data collection was granted by the Ministry of Education, Malaysia and the Kuala Lumpur Federal Territory Education Department. Parental consent is obtained for all children prior to participation, while verbal assent is also obtained from the children before the study begins.

The standard deviation (SD) of the BMI-for-age z-score obtained from a previous study (Madsen et al. 2009) is taken into consideration for the sample size calculation of this present trial. With the SD of the BMI z-score equal to 0.10, giving a detectable difference of 0.08, a sample size of around 25 children per group at 12 weeks would give 80 % power at the 0.05 significance level. The sample size was estimated using the following equation (Naing 2009):

$$ {\rm n} =[2 \upsigma^2/\Updelta^2]  ({\rm Z}_{\upalpha}+{\rm Z}_{\upbeta})$$

where n = estimated sample size, σ = standard deviation for BMI-score from previous study = 0.10, ∆ = detectable difference = 0.08, Z_α_ = significance level for two-sided test = 1.96, Z_β_ = 80 % power of study = 0.84.

The sample size required for this study is 25 per group. While taking into account a non-response rate of 50 %, the required sample size for each group is increased to 38; therefore, the total number of children needed is 76. Any children who wish to dropout are allowed to do so, with the reasons being noted and analysed.

### Rationale for the GReat-Child Trial

The GReat-Child trial is developed based on social cognitive theory (SCT). The SCT provides a theoretical foundation for identifying modifiable behaviours, as well as to structure behaviour-change interventions. Additionally, the SCT explains human behaviour in terms of a reciprocal relationship, the triadic and dynamic relationships in which behaviour patterns, personal characteristics and environmental influences interact (Baranowski et al. 2002). This theory has provided the conceptual framework for several multi-component interventions involving children and parents, which have resulted in positive dietary behaviour change (Baranowski et al. 2002).

The social cognitive theory concepts and applications for the present trial are shown in Table 1. Several SCT techniques will be applied in the present trial in order to assist the parents and children in raising their awareness of whole grain consumption and a healthy balanced diet, as well as to motivate the children and parents to make changes in order to manage childhood obesity. The theory constructs addressed in the GReat-Child trial include behaviour factors such as the behavioural capability (whole grain and healthy eating knowledge, as well as behavioural skills) and usual food choice (choice between pairs of foods); personal factors such as self-regulation (goal-setting), self-efficacy (confidence to carry out behaviours successfully) and outcome expectancies (preferences); as well as environmental factors consisting of the availability and accessibility of wholegrain foods and a healthy balanced diet (Burgess-Champouxt et al. 2007). The present trial is adapted from a combination of interventions named “Power of 3: Get Healthy with Wholegrain Foods” (Burgess-Champouxt et al. 2007) and “WATCH clinic” (Madsen et al. 2009).

**Table 1:** Social cognitive theory (SCT) concepts and application for GReat-Child trial

SCT constructs	Application	Learning activities
Behavioural domain		
Behavioural capability	Provided whole grain and healthy eating knowledge	Participation in whole-grain and healthy balanced diet quiz in six 30-min nutrition education classes Children were required to draw the healthy plate model on the blackboard Using food labels to identify foods as whole grain or refined grains Locating grain food group and grain food items on the Food Guide Pyramid Individual diet counselling for the parents to identify the whole-grain foods, as well as the advantages of whole grain consumption
Food choice	Served a variety of whole-grain foods on a daily basis	Tasting whole-grain foods for taste, appearance, texture and acceptance Researcher introducing variety of whole-grain food during school delivery and let the children familiarising with it Identifying whole-grain and healthy balanced diet’s labels during individual diet counselling for the parents
Personal domain		
Self-efficacy	Provided practical experiences that emphasised tasting, selecting and preparing whole-grain foods	Whole grain recipe booklet provided to parents during individual diet counselling, to help them prepare and serve whole-grain food at home Handy tips included in the whole grain booklet during individual diet counselling, to show the parents easier way to achieve the recommendation to eat half of the grains as whole grain Learning about menu planning for balanced diet during six 30-min nutrition education and individual diet counselling Reading food and nutrition labels on the packaging of whole-grain foods, to overcome barriers in selecting and identifying the whole-grain foods
Reinforcements	Children were rewarded and received praise when they correctly answer questions on whole-grain and healthy eating diet	Participation in whole-grain and healthy balanced diet quiz during nutrition education classes
Environmental domain		
Observational learning	Served a variety of whole-grain foods on a daily basis	Children observed how to prepare a convenient whole-grain breakfast Whole-grain food recipes provided to parents
Availability	Provided information to encourage the parents to increase availability of whole-grain foods and healthy diet at home	Individual diet counselling and booklet provided to advocate for consumption of more whole-grain foods, as well as balanced diet at home

## GReat-Child Trial Components

### Overview

An overview of the GReat-Child trial is shown in Table 2. This trial consists of three components addressing the behaviour, personal and environmental factors based on the SCT: (1) a family involvement component; (2) six 30-min nutrition education classes and (3) the school delivery of whole-grain foods. A quasi-experimental study design is utilized to increase the awareness and consumption of whole grain, modify the availability of whole-grain food by providing selected wholegrain foods every school day during break time, as well as to manage childhood obesity by reducing anthropometric measurements. Children from the intervention group received a 12-week intervention programme with another 6-month follow up. In contrast, the children in the control group do not receive any intervention activities, however, for ethical purposes, a health talk is conducted after the entire GReat-Child Trial is completed.

**Table 2:** The overview of GReat-Child trial

Components	Contents
Classroom nutrition education	Six 30-min nutrition education classes On fortnightly basis Employed simplified “Food Guide Pyramid” and “visual plate model” Module included: An overall food pyramid education Energy balance education A visual plate model An overall whole grain education Source and sampling of wholegrain foods Reading labels
Twelve-week school delivery of wholegrain foods	Provide opportunities for children to experience and accept wholegrain foods. On daily basis, during school-break time Selected wholegrain food: wholegrain bread, wholegrain ready-to-eat cereals and wholegrain biscuits
Family involvement	A session of individual diet counselling after the baseline data collection and prior to 12-week intervention Employed simplified “Food Guide Pyramid” and “visual plate model” Parents to increase the availability of wholegrain foods and balanced diet at home Encourage parental role modelling for consuming wholegrain foods and healthy diet Mother was invited to attend an in-depth interview session using a semi-structured telephone interview, at the conclusion of the GReat-Child Trial (9th month), to identify the obstacles they had encounter in increasing the wholegrain consumption

### Family involvement

This component includes a session of individual diet counselling with the parents prior to the 12-week intervention, just after the baseline data collection, which is based on the concept of the SCT (Table 1). Each parent is seen individually by a dietitian, and the dietary strategies used are the simplified “Food Guide Pyramid” and “visual plate model”. During the session, the parents is informed individually about their child’s BMI z-scores, percentage of body fat and waist circumferences, which are obtained from the baseline data collection. A dietary meal plan is given, based on modification of individual dietary recalls, which are also obtained from the baseline data collection. These are done to increase the parental awareness of childhood obesity, as well as to motivate them to monitor the anthropometric measurements and dietary intakes of their children. In addition, all parents is educated on whole-grain and healthy balanced diet recommendations based on the Malaysian Dietary Guidelines for Children and Adolescents (National Coordinating Committee on Food and Nutrition Ministry of Health Malaysia. Malaysian Dietary Guidelines for children and adolescents 2013). They are taught to read food labels, identify whole-grain foods, reduce portion size and increase physical activity. Additionally, the parents are encouraged to prepare wholegrain foods with a healthy balanced diet at home. Several handy tips are given to inform the parents of easier ways to achieve the guidelines of eating half of their grains as whole grains. The aims of this component are to: (1) increase the availability of whole-grain food at home; (2) encourage parental role modelling for consuming whole-grain foods and (3) promote a dialogue between the parents and the dietitian regarding the inclusion of whole-grain foods within the context of a healthy balanced diet. In addition, each parent receives written educational materials that contain whole grain recipes, handy tips for preparing whole-grain foods and healthy balanced diet recommendations, which are discussed during the individual diet counselling session.

### Classroom nutrition education classes

Six 30-min nutrition education classes based on the SCT (Table 1) are developed and delivered to the children who enrolled in the intervention group by a dietitian. A teacher attends each of the nutrition education classes to provide disciplinary assistance when needed. The intervention group receives the nutrition education classes on Wednesday afternoon at 1 p.m., on a fortnightly basis, after finishing their regular school lessons, and before they begin their compulsory after-school co-curriculum activity, which is implemented at 1.30 p.m. based on the recommendation of the Ministry of Education. This arrangement is made so that the delivery of the nutrition education will not affect the schedule, as well as to minimize any inconvenience to all parties concerned.

The classroom nutrition education component employs a simplified “Food Guide Pyramid” and “visual plate model” in order to provide guidance on how to obtain a balanced diet, with adequate whole grain intake. The aims of this nutrition education are to (1) improve knowledge on whole grains and balanced diets; (2) reduce intake of refined carbohydrates, and substituting with high-fibre whole grain and (3) improve attitudes and practices towards whole grain by learning to identify of whole-grain foods. The nutrition education module includes: (1) energy balance; (2) food pyramid; (3) a visual plate model; (4) whole grain; (5) source and sampling of whole-grain foods and (6) understanding food labels. At the end of each nutrition education class, the children participate in a quiz session to demonstrate their acquired knowledge regarding whole grain and healthy balanced diet, and those that answer the quiz correctly are rewarded with a stationery set. Each child also receives written educational materials that contain the plate model, dietary and whole grain recommendations that are discussed in the nutrition education classes.

### School delivery of wholegrain foods

For this component, whole-grain foods are delivered on a daily basis during the school break by a dietitian. The selected whole-grain foods, such as whole-grain ready-to-eat cereals, whole-grain bread and whole-grain biscuits are supplied to the children to replace any food that the children would normally eat during break time. This arrangement is made so that 100 % of the wholegrain food distributed would be eaten by the children under the dietitian’s supervision. All food items provided are bought by the researchers and are not sponsored by any food industry. For any children who are absent on a particular day, the whole-grain foods would be distributed the next day to the absent children. The absent child would take the extra whole-grain food home for consumption later, during the weekend. To make sure the children follow the instructions given, parents are reminded via phone calls or short messages on that particular weekend.

The school delivery of the whole-grain food component focuses on the inclusion of whole-grain food examples and activities that reflect the children’s lifestyles, preferences and culture, according to the concept of the SCT (Table 1). The goals of this component are to (1) offer the equivalent of one serving of whole-grain foods to the children by replacing refined-grain foods with whole grain counterparts and (2) provide the opportunity for the children to experience and accept a healthy new repertoire of whole grain alternatives in their diet. The selected whole-grain foods are convenient and nutrient-dense foods that do not require complicated preparation.

### Intervention outcome measurements

The children are assessed on three occasions, at the baseline, at the thirteenth week and at the ninth month. All the assessments are done by one investigator throughout the study to avoid the problem of inter-interviewer variations.

### Anthropometric measurement outcomes

The primary outcome measured is the BMI-for-age z-score, which is compared between the intervention and control groups. The body weight and height of each child are measured twice, according to standard procedures, using a calibrated Tanita digital scale Model SC-330 (Tanita Co., Tokyo, Japan) and SECA Bodymeter 217 (SECA GmbH & Co., Hamburg, Germany), respectively. The measurements are recorded to the nearest 0.1 kg and 0.1 cm, respectively. The body mass index (BMI) of each child is calculated by dividing the measured weight (kg) by the square of the height (m). The World Health Organization (WHO) BMI-for-age growth reference for children aged 5–19 years old (30) served as the standard reference for determining the nutritional status of the children. The z-scores for BMI-for-age (BAZ) are determined using the software WHO AnthroPlus version 1.0.3, and children are classified into overweight (BAZ +1SD to +2SD) and obese (BAZ > +2SD) categories. The percentage of body fat is measured twice by bioelectrical impedance using the TANITA digital scale Model 300GS (Tanita Co., Tokyo, Japan) and nearest to 0.1 %. The children stood bare footed on the scale, after the electrode of the scale is cleaned to remove any debris. Any objects that could interfere with the readings are removed from their pockets before the measurements are taken.

Waist circumferences (WC) of the children are measured twice, according to the standardised protocol by the National Health and Nutrition Examination Survey (NHANES), which assessed the WC just above the right iliac crest at the mid-axillary line (Centers for Disease Control and Prevention National Health and Nutrition Examination Survey 2000). The WC is measured to the nearest 0.1 cm by using a Lufkin tape model W606PM (Apex Tool Group, Maryland, USA). Finally, the reported values of the body weight, height, percentage of body fat and WC are the average values from the two readings taken.

### Nutrient intake outcomes

The nutrient intakes are assessed using three non-consecutive days’ 24-h diet recalls, consisting of 2 weekdays and 1 weekend day over the period of a week, a method which had been used by many national surveys and studies on children (School Nutrition Dietary Assessment 2007). They are completed through face-to-face interviews with each children using household measurements, portion sizes and estimated weights of the food consumed. Tableware items such as bowls, dishes, spoons and glasses in commonly-used sizes, food models and pictures of common foods are used to assess the food intake to enhance the portion sizes and the estimation of their respective weights. Pictures from the book, *Atlas of Food Exchanges and Portion Sizes* (Suzana et al. 2009), is used to help the child estimate their food portion sizes. Additionally, energy, macronutrient, micronutrient and dietary fibre intakes are determined from the 3-day diet recalls using Nutritionist Pro™ software (Axxya Systems, United States), based principally on the Nutrient Composition of Malaysian Food (Tee et al. 2005) and the food product labels. The children’s nutrient intake is compared with the Recommended Nutrient Intakes for Malaysia (RNI) (National Coordinating Committee on Food and Nutrition (NCFFN) 2005).

### Whole grain intake outcomes

The American Association of Cereal Chemists International (AACCI) definition of whole grain is applied in the present trial. According to the AACCI, whole grain includes cereal grains that consist of ground, cracker or intact grains, which incorporate all of the components of natural grain, including the endosperm, bran and germ (American Association of Cereal Chemists (AACC) International 2000). The components are present in the same relative proportions in whole grain cereals as they exist in the intact grain. For the purpose of the GReat-Child Trial, whole-grain foods are defined as foods made with at least one whole grain ingredient. All foods containing whole grain ingredients are included, regardless of the amount of whole grain they contained. The children who consumed a whole-grain food on at least one of the recall days are defined as whole grain consumers (Katcher et al. 2008). The amount of whole grain per 100 g in each of the whole-grain foods is estimated from the food label, or directly obtained from the food manufacturer. The whole grain values obtained per 100 g are then multiplied by the actual weight of the food consumed and divided by 100.

### Knowledge, attitudes and practices towards whole grain outcomes

The knowledge, attitudes and practices (KAP) towards whole grain among the children are assessed using a validated, guided, self-administered questionnaire. The details of the KAP towards the whole grain questionnaire, as well as the development and validation procedure have been previously published (Koo et al. 2016a). However, for the sake of completeness, a brief description is provided here. The questionnaire is constructed in the Malay language, and consists of four main sections: (1) demographic factors, (2) knowledge domain, (3) attitude domain and (4) practice domain. The demographic factors are intended to discover the demographic and whole grain consumption pattern. Whereas, the knowledge domain reflects the knowledge on general nutrition and whole grain information, including the food pyramid, source of carbohydrates, definition of whole grains, source of whole grains, nutritional content of whole grains and the benefits of whole grain consumption. The attitude domain is defined as the school children’s opinions and beliefs towards whole grain consumption, awareness and socio-cultural perspectives. Finally, the practice domain corresponds to the school children’s practice towards whole grain consumption, such as the frequency of the intake of whole grain ready-to-eat cereals, whole-grain bread, corn, whole-grain biscuits, oats, barley and brown rice.

### Physical activity measurement outcome

In the GReat-Child Trial, physical activity is measured using a pedometer, Digi-walker CW-701 (Yamax, Fukuyama, Japan), which measures step counts during the children’s waking hours over seven consecutive days for the intervention and control groups. The children are instructed to wear the pedometer on a waist belt, at all times, except while sleeping, swimming and showering. The pedometer has a memory recall to allow the investigator to recover the step counts, and a weighted average daily step count is calculated from the weekdays and weekend days. A minimum number of valid days (at least 3 weekdays and 1 weekend day) are used for the calculation of the average pedometer step counts (Laurson et al. 2008). The pedometer step counts are considered valid if the weighted average step counts are more than 1000 steps per day (Duncan et al. 2006), and the children wear the pedometer for at least 10 h per day (Laurson et al. 2008).

### In-depth interview outcomes

At the conclusion of the GReat-Child trial (9th month), mothers from the intervention group will be invited to participate in an in-depth interview after the second post-intervention, to identify the obstacles they had encountered in increasing whole grain consumption, as well as the changes in the attitudes and practices of their children. This session is important for investigating the sustainability of the present trial, and providing greater insights into the acceptance of whole grain among Malaysian children. Our unpublished preliminary data showed that majority of the children’s food (92.1 %) were prepared by their mother, which is line with the study of Campbell et al. (2007) that also showed that mothers played an important role in children’s dietary habits. Hence, the in-depth interviews involve only mother instead of both parents. The data is collected using semi-structured telephone interviews, and the entire interview conversations are audio-taped and transcribed verbatim. Field notes are taken during and immediately following each phone interview, with regards to the thoughts and ideas of each interviewee.

### Statistical analyses

Statistical analysis is conducted using the SPSS version 22.0 (IBM SPSS Statistics 2014). Data are entered, cleaned and checked before data analyses. Each variable is examined for normality distribution using the Kolmogorov–Smirnov test. Categorical data will be presented as number and percentage. Continuous data will be presented as mean and standard deviation or median and range. Association between categorical variables items and the two groups (intervention and control groups) are determined using Chi square test. The differences between intervention and control group across anthropometric measurements, pedometer step counts, whole grain and dietary intakes, as well as scores of KAP towards whole grain are determined using independent *T* test and Mann–Whitney U test. Analysis of covariance for repeated measures (ANCOVA) is performed to determine the intervention effects, such as the differences in anthropometric measurements, pedometer step counts, whole grain and dietary intakes, as well as scores of KAP towards whole grain between the intervention and control groups. Three models were examined, including (1) within group difference based on time; (2) between group differences regardless of time and (3) between group differences with regard to time. Baseline variables are considered as confounders in each models in order to prevent bias. Model assumptions including normality of the residuals, homogeneity of variance, compound symmetry and homogeneity of regression will be verified. P-value of less than 0.05 for a two-sided test is considered statistically significant.

## Discussion

The childhood obesity epidemic is one of the biggest current challenges for health policy; therefore, effective interventions to manage childhood obesity are needed. This, will in turn improve the health status of children and reduce the economic burden of obesity. A significant reduction in BAZ has been reported in a cross sectional study, which examined the relationship between the intake of whole grain and BAZ (Choumenkovitch et al. 2012); however, none of the experimental studies examined the effectiveness of whole grain consumption intervention in managing childhood obesity. To the best of our knowledge, this is the first multi-component, family-based intervention study focussing on whole-grain combined with healthy balanced diet to manage childhood obesity.

The essential elements of interventions are likely to include dietary modifications and nutrition education (Kirk et al. 2005). For example, one previous study demonstrated that when nutrition education was used as an intervention strategy, there was an improvement in nutrition knowledge, attitudes and eating behaviours (Prelip et al. 2011). Therefore, six 30-min nutrition education classes are included in the GReat-Child Trial, which aimed to improve the level of the KAP towards whole grains among the children. The duration of the nutrition education classes is only 30 min per session, as a previous study has shown that students typically have short attention span of only 10–15 min (Bunce et al. 2010).

Several studies from different countries and continents, including Malaysia (Koo et al. 2016b), Bahrain (Nadia and Parveen 2011) and Europe (Kleiman et al. 2012), have demonstrated that children lack dietary fibre and several micronutrients in their diets, despite having adequate calorie and macronutrient intakes. These studies also revealed that fibre insufficiency was apparently due to low consumption of vegetables, fresh fruits and other sources of fibre, such as whole grain, legumes and high fibre cereals, as observed in diet recalls. The micronutrient deficiencies may increase the risk of childhood obesity, due to fat deposition and chronic inflammation (Garcia et al. 2009); however, dietary cereals enriched with whole grains appeared to be more useful in the maintenance of adequate micronutrients and increasing the levels of fibre (Ortega et al. 2009). Therefore, the GReat-Child Trial is conducted with an emphasis on whole grain as a strategy to manage childhood obesity.

Low consumer awareness, confusion about the identification of whole grains, limited availability and disinclination to the taste of whole grain foods, texture and colour are the most habitually cited obstacles for the consumption of whole-grain foods (Burgess-Champouxt et al. 2006). According to Freeland-Graves and Nitzke (2007), the outcomes of an intervention are more likely to be positive if an organisation includes the activities that reflect the targetted school-aged children’s culture, lifestyle and preferences, as well as distribution of food samples in the intervention programs. Thus, to provide the opportunity for children to experience and embrace a healthy new repertoire of whole grain alternatives in their diets, the GReat-Child Trial offered one serving of whole-grain foods to the children on a daily basis, by replacing refined-grain foods with various types of wholegrain counterparts. In addition, the present trial involved greater contact time with the children and included the parents, unlike a quasi-experimental trial from the US which involved school cafeteria workers, and aimed to increase the whole grain consumption among children (Burgess-Champouxt et al. 2007). Modifications are made in order to place the parents as the main agents of change, because this approach was successful in a previous Malaysian randomized controlled trial that aimed to manage childhood obesity (Wafa et al. 2011).

Social marketing and recipe modification strategies had been applied in previous interventions to successfully modify fat intake (Ellison et al. 1989), vegetable and fruit intakes (Reynolds et al. 2000), as well as intake of low-fat milk (Wechsler et al. 1998). The outcomes of these interventions showed improvements in consumption of low-fat milk, low-fat diet, vegetables and fruits due to the food preparation practices, where these interventions provided them with convenient, palatable and easy to prepare options. In the present study, the selected whole-grain foods included whole-grain bread, whole-grain ready-to-eat cereals and whole-grain biscuits, because these were convenient and nutrient-dense, and did not require complicated preparation. Encouragingly, a recent study of 10–11 years old Malaysia schoolchildren in Kuala Lumpur reported that bread, biscuits and ready-to-eat cereals were the most commonly eaten foods at breakfast (Koo et al. 2015b). In addition, ready-to-eat cereal consumption showed an inverse association with BMI and waist circumferences (Koo et al. 2014).

The main strength of the GReat-Child Trial is that it represents a novel approach to examine the effectiveness of intervention with whole-grain and healthy balanced diet in managing childhood obesity. We anticipate that this study will not only reveal if an intervention focussing on consumption of whole grain in combination with healthy balanced diet will be effective, but that it will also provide greater insights into the acceptance of whole grain among Malaysian children. Longer duration of intervention may produce better outcomes in managing childhood obesity, but such interventions are much less likely to be practical (Wafa et al. 2011). The efficacy of the GReat-Child Trial described in this paper is not conclusive, as the study is still on-going, and further outcomes will be published in the near future.

## Conclusion

In conclusion, the GReat-Child Trial is the first multi-component, family-based intervention focussing on whole-grain consumption with healthy balanced diet for managing childhood obesity. The novel approach to obesity intervention, which is based on systematic reviews and clinical management guidelines for childhood obesity, provides evidence of the efficacy of whole grain intervention in managing childhood obesity. Furthermore, it will provide better understanding of the acceptance of whole grain among Malaysian children in particular and among Asian children in general.

## References

[CR1] American Association of Cereal Chemists (AACC) International (2000). Whole grain definition. Cereal Foods World.

[CR2] Anand SS, Davis AD, Ahmed R, Jacobs R, Xie C, Sowden J, Atkinson S, Blimkie C, Brouwers M, Morrison K, de Koning L, Gertein H, Yusuf S, Investigotars Share-AP Action (2007). A family-based intervention to promote healthy lifestyle in an aboriginal community in Canada. Can J Public Health.

[CR3] Baranowski T, Perry CL, Parcel GS (2002) How individuals, environments and health behavior interact: social cognitive theory. Health Behavior and Health Education: Theory, Research and Practice, 3rd ed 165–184

[CR4] Bellisle F, Hebel P, Colin J, Reye B, Hopkins S (2014). Consumption of whole grains in French children, adolescents and adults. Br J Nutr.

[CR5] Ben-Sefer E, Ben-Natan M, Ehrenfeld M (2009). Childhood obesity: current literature, policy and implications for practice. Int Nurs Rev.

[CR6] Bunce DM, Flens EA, Neiles KY (2010). How long can students pay attention in class? A study of student attention decline using clickers. J Chem Educ.

[CR7] Burgess-Champouxt TL, Marquart L, Vickers Z, Reicks M (2006). Perception of children, parents and teachers regarding whole-grain foods, and implication for a school-base intervention. J Nutr Educ Behav.

[CR8] Burgess-Champouxt TL, Chan HW, Rosen R, Marquart L, Reicks M (2007). Healthy whole-grain choices for children and parents: a multi-component school-based pilot intervention. Public Health Nutr.

[CR9] Campbell KJ, Crawford DA, Salmon J, Carver A, Garnett SP, Baur LA (2007). Associations between the home food environment and obesity promoting eating behaviors in adolescence. Obes.

[CR10] Centers for Disease Control and Prevention (2009) Childhood overweight and obesity consequences. http://www.cdc.gov/obesity/childhood/consequences.html

[CR11] Centers for Disease Control and Prevention National Health and Nutrition Examination Survey (2000) Anthropometry Procedures Manual 3-30.3-31

[CR12] Choumenkovitch SF, McKeown NM, Tovar A, Hyatt RR, Kraak VI, Hastings AV, Herzog JB, Economos SD (2012). Whole grains consumption is inversely associated with BMI z-score in rural school-aged children. Public Health Nutr.

[CR13] Davison KK, Jurknowski JM, Li K, Kranz S, Lawson HA (2013). A childhood obesity intervention developed by families for families: results from a pilot study. Int J Behav Nutr Phys.

[CR14] Duncan JS, Schofield G, Duncan EK (2006). Pedometer-determined physical activity and body composition in New Zealand children. Med Sci Sport Exerc.

[CR15] Ellison RC, Capper AL, Goldberg RJ (1989). The enviromental component: changing school foodservice to promote cardiovascular health. Health Educ Quart.

[CR16] Fernandez-Sanchez A, Madrigal-Santillan E, Bautista M, Esquivel-Soto J, Morales-Gonzalez A, Esquivel-Chirino C, Durante-Montiel I, Sanchez-Rivera G, Valadez-Vega C, Morales-Gonzalez JA (2011). Inflammation, oxidative stress, and obesity. Int J Mol Sci.

[CR17] Freeland-Graves J, Nitzke S (2007). Position of the American Dietetic Association: total diet approach to communication food and nutrition information. J Acad Nutr Diet.

[CR18] Garcia OP, Long KZ, Rosado JL (2009). Impact of micronutrient deficiencies on obesity. Nutr Rev.

[CR19] Golan M (2006). Parents as agents of change in childhood obesity—from research to practice. Int J Pediatr Obes.

[CR20] Graham D, Appleton S, Rush EMS, McLennan S, Reed P, Simmons D (2008). Increasing activity and improving nutrition through a schools-based programme: project Energize. Design, programme, randomization and evaluation methodology. Public Health Nutr.

[CR21] Gupta N, Chin MK, Yang JZ, Balasekaran G, Chia M, Robert N, Girandola Christopher RE, Mok MMC (2010). Obesity prevention in Singapore: collaborative efforts among government, health professionals and the community. Asian J Exerc Sport Sci.

[CR22] Harnack L, Walters SA, Jacobs DR (2003). Dietary intake and food sources of whole grains among US children and adolescents: data from the 1994-1996 Continuing Survey of Food Intakes by Individuals. J Am Diet Assoc.

[CR23] Health Promotion Board Singapore (2010) National Nutrition Survey. Research & Strategic Planning Division. http://www.hpb.gov.sg/HOPPortal/content/conn/HOPUCM/path/Contribution%20Folders/uploadedFiles/HPB_Online/Publications/NNS-2010.pdf

[CR24] Institute of Public Health (IPH). National Health and Morbidity Survey (NHMS) (2011) Non-communicable diseases, Ministry of Health Malaysia. Vol II

[CR25] James J, Thomas P, Kerr D (2007). Preventing childhood obesity: two year follow-up results from the Christchurch obesity prevention programme in schools (CHOPPS). Br Med J.

[CR26] Katcher HI, Legro RS, Kunselman AR, Gillies PJ, Demers LM, Bagshaw DM, Kris-Etherton PM (2008). The effects of whole grain-enriched hypocaloric diet on cardiovascular disease risk factors in men and women with metabolic syndrome. Am J Clin Nutr.

[CR27] Kipping RR, Jago R, Lawlor DA (2006). Obesity in childhood overweight and obesity. Int J Pediatr Obes.

[CR28] Kirk S, Scott BJ, Daniels SR (2005). Pediatric obesity epidemic: treatment options. J Am Diet Assoc.

[CR29] Kleiman S, Ng S, Popkin B (2012). Drinking to our health: can beverage companies cut calories while maintaining profits?. Obes Rev.

[CR30] Knowlden AP, Sharma M (2012). Systematic review of family and home-based interventions targeting paediatric overweight and obesity. Obes Rev.

[CR31] Koo HC, Suriyani MY, Ruzita AT (2014). Association between consumption of ready-to-eat cereal and anthropometric status among school children in Kuala Lumpur, Malaysia. Mal J Nutr.

[CR32] Koo HC, Poh BK, Ruzita AT (2015). Assessment of knowledge, attitude and practice towards whole grain among children aged 10 and 11 years in Kuala Lumpur, Malaysia. Int J Food Sci Nutr Diet.

[CR33] Koo HC, Siti Nurain AJ, Ruzita AT (2015). Breakfast eating pattern and ready-to-eat cereals consumption among schoolchildren in Kuala Lumpur. Mal J Med Sci.

[CR34] Koo HC, Poh BK, Ruzita AT (2016). Development, validity and reliability of a questionnaire on knowledge, attitude and practice (KAP) towards whole grains among primary school children in Kuala Lumpur, Malaysia. Int Food Res J.

[CR35] Koo HC, Poh BK, Lee ST, Chong KH, Bragt MCE, Ruzita AT (2016) Are Malaysian children achieving dietary guideline recommendation? Asia Pac J Public Health: in press 10.1177/1010539516641504 27073200

[CR36] Laurson KR, Eisenmann JC, Welk GJ, Wickel EE, Gentile DA, Walsh DA (2008). Combined influence of physical activity and screen time recommendations on childhood overweight. J Pediatr.

[CR37] Linberg SM, Adams AK, Prince RJ (2012). Early predictors of obesity and cardiovascular risk among American Indian children. Matern Child Health J.

[CR38] Madsen KA, Garber AK, Mietus-Snyder ML, Orrell-Valente JK, Tran CT, Wlasiuk L, Matos RI, Neuhaus J, Lustig RH (2009). A clinic-based lifestyle intervention for pediatric obesity: efficacy and behavioral and biochemical predictors of response. J Pediatr Endocrinol Metab.

[CR39] Maki KC, Beiseigel JM, Jonnalagadda SS, Gugger CK, Reeves MS, Farmer MV, Kaden VN, Rains TM (2010). Whole-grain ready-to-eat oat cereal, as part of dietary program for weight loss, reduced low-density lipoprotein cholesterol in adults with overweight and obesity more than a dietary program including low-fibre control foods. J Am Diet Assoc.

[CR40] McKeown NM, Yoshida M, Shea MK, Jacques PF, Lichtenstein AH, Rogers G, Booth SL, Saltzman E (2009). Whole-grain intake and cereal fibre are associated with lower abdominal adiposity in older adults. J Nutr.

[CR41] McMillan JH (2007). Randomized field trials and internal validity: not so fast my friend. Pract Assess Res Eva.

[CR42] Nadia G, Parveen R (2011). Energy and macronutrient intake and dietary pattern among school children in Bahrain: a cross-sectional study. Nutr J.

[CR43] Naing NN (2009). Sample size determination in experimental studies. A practical guide on determination of sample size in health sciences research.

[CR44] National Coordinating Committee on Food and Nutrition (NCFFN) (2005) Recommended Nutrient Intakes for Malaysia. Ministry of Health Malaysia, Kuala Lumpur. http://www2.moh.gov.my/images/gallery/rni/insert.pdf

[CR45] National Coordinating Committee on Food and Nutrition Ministry of Health Malaysia. Malaysian Dietary Guidelines for children and adolescents (2013). http://www.moh.gov.my/images/gallery/Garispanduan/MDG%20Children%20and%20Adolescents%20Summary.pdf

[CR46] Niemeier B, Hektner JM, Enger KB (2012). Parent participation in weight-related health interventions for children and adolescents: a systematic review and meta-analysis. Prev Med.

[CR47] Norimah AK, Koo HC, Hamid Jan JM, Mohd Nasir MT, Tan SY, Mahenderan A, Nurliyana AR, Thielecke F, Hopkis S, Ong MK, Ning C, Tee ES (2015). Whole grain intakes in the diets of Malaysian children and adolescents—findings from the MyBreakfast Study. PLoS ONE.

[CR48] Ortega RM, Am Lopez-Sobaler, Andres P, Rodriguez-Rodriguez E, Aparicio A, Bermejo LM (2009). Increasing consumption of breakfast cereal improves thiamine status in overweight/obese women following a hypocaloric diet. Int J Nutr Food Sci.

[CR49] Perry CL, Luepker RV, Murray DM, Kurth C, Mullis R, Crockett S, Jacobs DR (1998). Parent involvement with children’s health promotion: the Minnesota home team. Am J Public Health.

[CR50] Prelip M, Slusser W, Thai CL, Kinsler J, Erausquin JT (2011). Effects of a school-based nutrition program diffused throughout a large urban community on attitudes, beliefs and behaviors related to fruit and vegetable consumption. J Sch Health.

[CR51] Reilly JJ (2006). Trackling the obesity epidemic: new approaches. Arch Dis Chil.

[CR52] Reynolds KD, Franklin FA, Blinkley D (2000). Increasing the fruit and vegetable consumption of fourth-graders: results from the High 5 project. Prev Med.

[CR53] School Nutrition Dietary Assessment Study-III: Volumn II: Student participation and dietary intakes by Gordan A, McKinney P (2007) US Department of Agriculture, Food dan Nutrition Service, Office of Research, Nutrition and Analysis

[CR54] Sharifah WW, Nur Hana H, Ruzita AT, Rosless R, Reilly J (2011). The Malaysian childhood obesity treatment trial (MASCOT). Mal J Nutr.

[CR55] Suzana S, Noor Aini MY, Shanita SN (2009). Atlas of food exchanges and portion sizes.

[CR56] Tee ES, Noor MI, Azudin MN (2005). Nutrient composition of Malaysian foods.

[CR57] Theodore LA, Bray MA, Kehle TJ (2009). Introduction to the special issue: childhood obesity. Psychol Sch.

[CR58] Wafa SW, Talib RA, Hamzaid NH, McColl JH, Rajikan R, Ng LO, Ramli AH, Reilly JJ (2011). Randomized controlled trial of a good approach to treatment of childhood obesity in Malaysia: Malaysian Childhood Obesity Treatment Trial (MASCOT). Int J Pediatr Obes.

[CR59] Wechsler H, Basch CE, Zybert P, Shea S (1998). Promoting the selection of low-fat milk in elementary school cafeterias in an inner-city Latino community: evaluation of an intervention. Am J Public Health.

[CR60] World Health Organisation (WHO) (2007) Growth reference 5-19 years. http://www.who.int/growthref/who2007bmiforage/En/index.html

[CR61] Ye EQ, Chacko SA, Chou EL, Kugizaki M, Liu S (2012). Greater whole-grain intake is associated with lower risk of type 3 diabetes, cardiovascular disease and weight gain. J Nutr.

